# The effect of educational intervention on efficacy of 1% permethrin shampoo and 4% dimeticone lotion to treat head lice infestation using propensity score matching (PSM)

**DOI:** 10.1186/s12879-024-09029-1

**Published:** 2024-01-30

**Authors:** Eslam Moradi-Asl, Abedin Saghafipour, Amir Hamta, Zahra Taheri-Kharameh, Malek Abazari, Shabnam Asghari Jajin

**Affiliations:** 1 Arthropod-Borne Diseases Research Center , Ardabil University of Medical Sciences, Ardabil, Iran; 2 Department of Public Health, School of Public Health, Ardabil University of Medical Sciences, Ardabil, Iran; 3 Department of Public Health, Faculty of Health, Qom University of Medical Sciences, Qom, Iran; 4 Department of Family and Community Medicine, School of Medicine, Qom University of Medical Sciences, Qom, Iran; 5 Spiritual Health Center, Department of public Health, School of Health, Qom University of Medical Sciences, Qom, Iran; 6 Department of Medical Entomology, School of Public Health, Tehran University of Medical Sciences, Tehran, Iran

**Keywords:** Lice infestations, Pediculus, Health Education, Propensity score

## Abstract

**Background:**

Head lice are a main public health problem and the most important human ectoparasites and the use of pediculicides is the most common way to control it. One of the possible causes of treatment failure is the lack of improper application of pediculicide. The aim of this study was to assess the effect of education on efficacy of 1% permethrin or 4% dimeticone lotion to treat head lice infestation.

**Methods:**

This quasi-experimental study included 100 individuals with head lice infestation from comprehensive urban health centers in Ardabil as the intervention group, and 400 individuals from East Azerbaijan and West Azerbaijan provinces as the control group, from April to March 2019. The data collection tools included a demographic questionnaire and an examination recording sheet, which documented the presence of adult lice or nits. Due to the inability to perform random assignment and control for numerous observed covariates, propensity score matching (PSM) was used.

**Results:**

The outcome of treatment included elimination of head lice infestation on is 7, and in the case of recurrence, it was considered on days 14 and 30 after treatment. The results showed that the educational intervention program had a significant positive effect on the efficacy of both treatments. The likelihood of improvement was approximately three times greater in the intervention group compared to the control group.

**Conclusion:**

Participants who received the training intervention (OR = 3.29; CI 95%: 2.21–4.88) were more likely to have a successful treatment than control group. In the case of providing proper training on the use of pediculicides and observing hygiene tips to patients with pediculosis, could help to successful treatment of pediculosis.

## Background

Head lice (*Pediculus humanus capitis*) belong to the Phylum Arthropoda, Class Insecta, Order Phthiraptera, and it is considered as compulsive ectoparasitis of mammals, including humans and it is transmitted mainly by direct contact with the hair of an infected person [1]. Pediculosis, which results from head lice infestation in humans, is common in all parts of the world, but is more prevalent in places with high population densities [2]. Its prevalence in developing countries has been reported at 40%. The prevalence of head lice infestation in different regions of Iran is relatively high [3]. Head lice infestation can lead to feelings of inferiority, psychological distress, depression, insomnia, academic failure, stigma, secondary infestations, hair loss, and allergies [4]. One of the ways to prevent head lice is to follow personal hygiene, take regular baths and avoid using other people’s personal belongings [5]. Many therapies have been studies and evaluated for head lice treatment in the world and in Iran [6]. At the present time, in Iranian health centers, two products of 1% Permethrin or 4% dimeticone are routinely used to treat head lice. According to results of a study, the use of two anti-lice insecticides, permethrin% 1 shampoo and dimethicone 4% lotion, which are commonly used in Iran based on the recommendation of the Center for Management of Infectious Diseases, had a relatively equal effect on the treatment of people with head lice infestation [6]. While pediculicides can effectively kill adult head lice, they are often less effective at killing nits (lice eggs), and some nits may hatch after the incubation period and mature into adult lice, thus perpetuating the infestation cycle. In addition to these biological factors, there are also several other factors that can contribute to the recurrence of lice infestations. These may include frequent infestations in certain populations or regions, differences in infestation density between different communities or cities, variations in parental education levels and socio-economic status, the potential for drug resistance to treatments, and differences in the quality and effectiveness of patient education and prevention strategies. Taken together, these factors highlight the complex nature of lice infestations and the need for a multifaceted approach to their prevention and control [7]. Pediculicides may be more effective in treating pediculosis if used properly.

Several studies have evaluated the efficacy of these treatments, but few have investigated the effect of educational interventions on their effectiveness. A study conducted in Iran found that an educational intervention program improved the efficacy of 1% permethrin shampoo in treating head lice infestation in primary school students. The study showed that the efficacy of the treatment increased from 53.7 to 75.5% after the intervention [8]. Another study conducted in Turkey evaluated the effect of an educational intervention on the efficacy of 4% dimeticone lotion in treating head lice infestation in school children. The study showed that the educational intervention significantly improved the efficacy of the treatment from 77.8 to 98.1% [9].

Overall, the literature suggests that educational interventions can improve the efficacy of 1% permethrin shampoo and 4% dimeticone lotion in treating head lice infestation. PSM can be used to evaluate the effectiveness of these treatments and reduce bias in observational studies. Further research is needed to determine the optimal educational interventions and their impact on treatment outcomes in different populations. Due to the high prevalence of head lice in recent years in in the northwest of the Iran such as Ardabil province (with a population of about one million and four hundred thousand people) [10] and the increasing trend in the use of anti-insect solutions and imposing a heavy cost on the patient care system, this study aims to evaluate the effect of educational intervention on the efficacy of two common anti-lice insecticides in Iran, namely 1% permethrin shampoo and 4% dimethicone lotion in the treatment of head lice infestation using statistical method of propensity score matching (PSM).

## Materials and methods

### Design and participants

In this quasi-experimental study, the efficacy of common anti-insect solutions used in the treatment of head lice (Permethrin 1% shampoo or 4% dimethicone lotion) prescribed routinely by Ministry of Health, was done on 100 subjects with head lice infestation in comprehensive urban health centers of Ardabil province (intervention group) and 400 subjects of East Azerbaijan and West Azerbaijan (control group) provinces from April to March 2019 using census method. The inclusion criteria for this study were: (1) presence of head lice and lice eggs, (2) provision of informed consent by the participant to participate in the study, and (3) having a record of head lice infestation in the health care centers of Ardabil city and the exclusion criteria were: (1) not having used any anti-lice products in the two weeks prior to the study, (2) not willing to use the specified shampoo and lotion during the study, (3) having a known allergy to permethrin shampoo and dimethicone lotion, and (4) being pregnant or breastfeeding. The two provinces of Azerbaijan and Ardabil, from which case and control samples have been selected, are completely similar in terms of culture, customs, lifestyle, economic and social status. The number of subjects for each treatment method in all urban and rural areas was equal, so the equal distribution of socio-economic confounding factors in response to treatment was ensured. The most important confounding factor in therapeutic effect was the possibility of re-infestation of people who are around the infected person after the start of the trial and treatment, which could potentially appear in the role of reducing the therapeutic effect. To overcome it, all home contact cases, without considering that infestation was diagnosable or non-diagnosable, were treated simultaneously.

### Intervention and outcome measure

In the intervention group, before prescribing the shampoos, the correct method of using these anti-lice shampoos was practically taught by the medical entomology experts. Subjects in intervention group received 30 min of training. The training consisted of the head lice and ways of transmitting the disease (10 min), the pediculicide such as 1% permethrin and 4% dimeticone lotion (10 min) and how to use them properly in the treatment of head lice (10 min) using role-playing method. However, the control group in both East and West Azerbaijan provinces were treated routinely. Each treatment included using 1% permethrin shampoo twice or 4% dimethicone lotion according to the instructions. The outcome of the treatment included removal of adult human head lice, nymphs, and nits in confirmed human cases at 14 and 30 days after starting treatments were considered. Data were collected through a researcher-made questionnaire and through observation and examination. To assess reliability, two examiners was used the sheet to document lice or nit presence in the same individuals. The level of agreement between the examiners was measured using Cohen’s kappa. The kappa value was equal to 1 indicated good reliability of the examination recording sheet.

The training sessions were conducted in person, with each group consisting of 10 individuals. Notably, the cost of treatment was completely covered for all participants. In the control group, individuals received standard treatment as per the Ministry of Health’s guidelines, while also benefiting from face-to-face training prior to the commencement of treatment. The questionnaire was administered and completed by the examiner.

### Data analysis

The statistical analysis used in this study included PSM and generalized estimating equations (GEE).

**Fig. 1:**
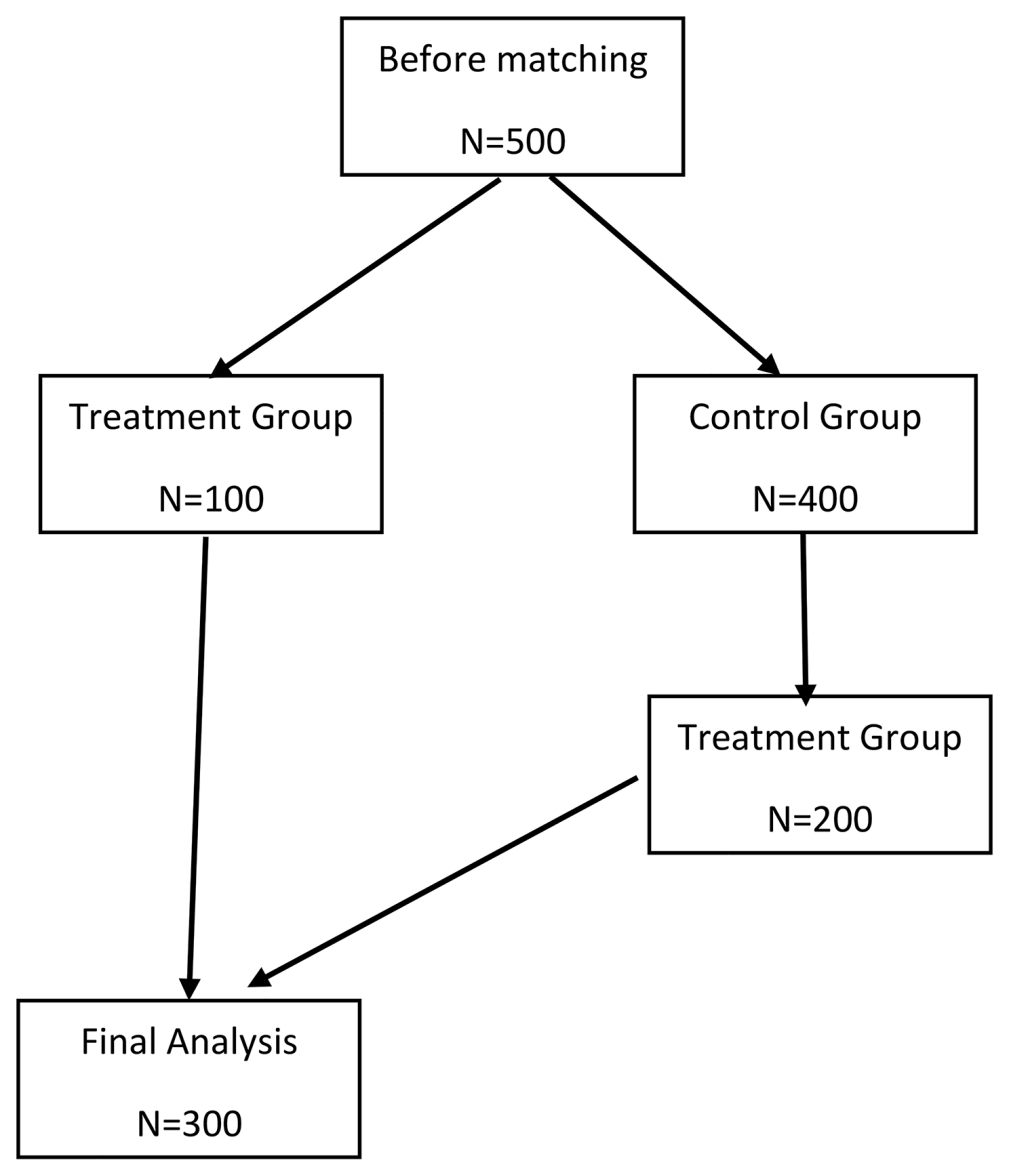
Study Follow Diagram

PSM is a statistical technique used to reduce potential bias in observational studies by creating matched groups of individuals with similar characteristics. In this study, PSM was used to balance the distribution of covariates between the intervention and control groups, since random assignment was not possible [11, 12].. In this study by using PSM confounding variables and treatment selection were controlled. Based on PSM multidimensional pretreatment confounder covariates transformed to one-dimensional variable and as a result similarity in distribution of covariates in both groups is happened [13]. Next the propensity score was calculated, and matching was done. All 100 patients in case group were matched by applying by1:2 PSM employing nearest neighbor method. Prognostic score based balanced was used in order to evaluating bias reduction in PSM. The most popular matric balance measure prognostic score, standardized mean difference (SMD), has been used. This statistic is used to examine the balance of confounders between intervention and control group [14]. SMD greater than 0.1 shows the significant dissimilarity [15]. The strength of SMD is sample size does not have influence on it [16]. Figure 1 shows study follow diagram. In the next step matched data, since head lice infestation each individual was investigated three times and measurements are dependent, for considering dependency, generalized Estimating Equations (GEE) was applied [17]. All statistical analysis were done using R version 3.6.3 software.

## Result

In total, 100 subjects in intervention group and 400 subjects in control group completed the study. The result showed before applying PSM the mean age of intervention and control group was significantly different. The age of individual in intervention group was $16.18\pm 12.61$ and in control group it was $13.09\pm 10.21$ (*P*<0.05) and SMD for age of participants 0.27 was obtained. Conspicuously SMD showed the distribution of age is too different to compare, while after applying PSM the age of individuals in both group was not statistically significant any more ($16.18\pm 12.61$ vs. $16.34 \pm 12.52$ in intervention and control groups respectively; *P*-value=0.92). The comparison of characteristics of both group before and after applying PSM are presented in Table 1. As well as the Table 2 illustrates the outcomes of head lice treatment using 1% permethrin and 4% dimethicone lotion in both the intervention and control groups.

**Table 1:** Individual characteristics before and after matching

variables	Before PSM count (Percent)					After PSM count (Percent)			
	Control group, *n* = 400	Treatment Group, *n* = 100	SMD	P		Control group, *n* = 200	Treatment Group, *n* = 100	SMD	P
**Sex**									
1	105 ( 26.2)	25 ( 25.0)	0.029	0.899		51 ( 25.5)	25 ( 25.0)	0.012	> 0.999
2	295 ( 73.8)	75 ( 75.0)				149 ( 74.5)	75 ( 75.0)		
**Size of hear**									
1	127 ( 31.8)	28 ( 28.0)	0.109	0.617		67 ( 33.5)	28 ( 28.0)	0.121	0.490
2	167 ( 41.8)	41 ( 41.0)				69 ( 34.5)	41 ( 41.0)		
3	106 ( 26.5)	31 ( 31.0)				64 ( 32.0)	31 ( 31.0)		
Infestation rate									
1	144 ( 36.0)	30 ( 30.0)	0.207	0.161		62 ( 31.0)	30 ( 30.0)	0.071	0.844
2	188 ( 47.0)	45 ( 45.0)				94 ( 47.0)	45 ( 45.0)		
3	68 ( 17.0)	25 ( 25.0)				44 ( 22.0)	25 ( 25.0)		
**Type of treatment**									
1% permethrin shampoo	206 ( 51.5)	50 ( 50.0)	0.030	0.876		92 ( 46.0)	50 ( 50.0)	0.080	0.595
4% dimeticone lotion	194 ( 48.5)	50 ( 50.0)				108 ( 54.0)	50 ( 50.0)		

**Table 2:** Head lice treatment results with 1% permethrin and 4% dimeticone lotion in intervention and control group

Efficacy of treatment	1% permethrin shampoo						4% dimeticone lotion					
	7 day (*n* = 142)		14 day (*n* = 83)		30 day (*n* = 46)		7 day (*n* = 158)		14 day (*n* = 116)		30 day (*n* = 86)	
	Intervention *n* = 50	Control *n* = 92	Intervention *n* = 14	Control *n* = 69	Intervention *n* = 8	Control *n* = 38	Intervention *n* = 50	Control *n* = 108	Intervention *n* = 34	Control *n* = 82	Intervention *n* = 21	Control *n* = 65
Cured	36(72.0)	23(25)	6(42.9)	31(44.9)	4(50)	13(34.2)	16(32.0)	26(24.1)	13(38.2)	17(20.7)	15(71.4)	9(8.3)
Failure	14(28.0)	69(75.0)	8(57.1)	38(55.1)	4(50)	25(65.8)	34(68.0)	82(75.9)	21(61.8)	65(79.3)	6(28.6)	56(86.2)

GEE analysis showed there is significant difference in evaluating head lice over time. Participants who received the training intervention (OR = 3.29; CI 95%: 2.21–4.88) were more likely to have a successful treatment. In addition, individuals who utilized 1% permethrin shampoo demonstrated a significantly higher likelihood of improvement (OR = 0.39; CI 95%: 0.27–0.57) in comparison to those who relied on 4% dimethicone lotion. (Table 3).

**Table 3:** Summarized GEE analysis on the results

	Parameter	B	Std. Error	OR	95% Wald Confidence Interval for Exp(B)		*P*-value
					Lower	Upper	
Group	Intervention	1.191	0.2014	3.291	2.218	4.885	< 0.001
	Control Reference	-	-	-	-	-	-
Shampoo	1% permethrin shampoo	-0.931	0.1916	0.394	0.271	0.574	< 0.001
	4% dimeticone lotion Reference	-	-	-	-	-	-
Dandruff	4	0.441	0.4482	1.554	0.646	3.74	0.325
	3	0.067	0.2567	1.069	0.646	1.768	0.796
	2	0.26	0.2268	1.297	0.832	2.023	0.251
	1 Reference	-	-	-	-	-	-
Hair volume	3	0.842	0.2883	2.32	1.319	4.082	0.004
	2	0.259	0.2472	1.295	0.798	2.103	0.295
	1 Reference	-	-	-	-	-	-

B: Regression coefficient

## Discussion

The present study aimed to evaluate the effect of an educational intervention program on the efficacy of 1% permethrin shampoo and 4% dimeticone lotion in treating head lice infestations, using PSM. The study findings revealed that the educational intervention program had a significant positive effect on the efficacy of the two treatments, as measured by the eradication rate of head lice and nits. Based on the results of the present study, the educational intervention showed a significant difference in the test group, but in the control group, this difference was not significant. In other words, teaching the way of using pediculicides properly and health tips were effective in treating and reducing recurrence. Several studies have been conducted in the treatment of head lice infestation [18–19]. Most studies have compared different treatments [19–21], and several studies have been conducted on the effectiveness of training programs on preventing pediculosis capitis [22–23]. A study conducted in Iran found that an educational intervention program improved the efficacy of 1% permethrin shampoo in treating head lice infestation in primary school students [8]. Another study in Turkey showed that an educational intervention significantly improved the efficacy of 4% dimeticone lotion in treating head lice infestation in school children [9]. In a study conducted by Zareban, the health education program had a positive effect on reducing head lice infestation, and after educational intervention, the rate of infestation in the experimental group decreased by 17.6%%, but it did not difference in the control group [24]. In the study conducted by Chegini (2017), mothers received group discussion on the preventive behaviors of pediculosis and it led to a reduction in the incidence of pediculosis in their daughters [23]. However, in the study conducted by Eftekhari (2017), educational intervention is effective in promoting preventive behaviors against pediculosis on female elementary school students [22]. Similarly, in another study, the prevalence of pediculosis was significantly reduced after implementing educational interventions in the school [25].

According to our study, individuals who opted for 1% permethrin shampoo exhibited a significantly greater likelihood of improvement compared to those who relied on 4% dimethicone lotion. Previous research has also shown that 4% dimeticone lotion effectively treats head lice infestations, while causing fewer irritant reactions than alternative treatments [26]. However, another study suggests that the efficacy of these treatments does not significantly differ from one another [6].

The use of PSM in this study helped to reduce the bias that could have been introduced by differences between the intervention and control groups. By matching subjects based on their propensity score, the groups were more comparable and differences in outcomes could be attributed more reliably to the educational intervention program. A major strength of this study is the use of PSM, which helped to reduce the potential for bias in the study design. PSM is a well-established statistical technique that can improve the validity of observational studies by balancing the distribution of confounding variables between treatment groups.

The limitation of this study is the potential for selection bias, as the study participants were recruited from health care centers in Ardabil city. This may limit the generalizability of the findings to other populations. Additionally, the study did not assess the long-term efficacy of the treatments or the durability of the effect of the educational intervention program beyond the duration of the study.

The findings of the present study suggest that educational interventions can be a valuable addition to the treatment of head lice infestations. Providing information on proper application techniques, the importance of adherence to the treatment regimen, and other related aspects could enhance the efficacy of the treatments. This, in turn, could reduce the risk of treatment failure and the spread of head lice infestations in the community.

In conclusion, this study provides evidence that an educational intervention program can improve the efficacy of 1% permethrin shampoo and 4% dimeticone lotion in treating head lice infestations. The use of PSM helped to mitigate potential bias in the study design, and the results are consistent with previous research on the value of educational interventions in this area. Further research is needed to evaluate the optimal content and delivery of educational interventions for head lice infestations, as well as their impact on treatment outcomes in different populations.

## Data Availability

All data generated or analyzed during this study are included in this published article.
